# 841. Survey of Current HIV and HCV Policies and Practices in Prisons and Jails Serving High-Risk Geographic Hotspots

**DOI:** 10.1093/ofid/ofab466.1037

**Published:** 2021-12-04

**Authors:** Sugi Min, Jimin Shin, Brendan Jacka, Lauri Bazerman, Ank E Nijhawan, Curt Beckwith

**Affiliations:** 1 Columbia University Irving Medical Center, New York, New York; 2 The Miriam Hospital, Providence, Rhode Island; 3 Brown University School of Public Health, Providence, Rhode Island; 4 University of Texas Southwestern, Dallas, TX; 5 Brown University School of Medicine, Providence, RI

## Abstract

**Background:**

The goal of the U.S. “Ending the HIV Epidemic” (EHE) initiative is to reduce new HIV infections by 90% within 10 years by focusing resources on high-risk geographic “hotspots.” (Figure 1). The criminal justice system bears a disproportionate burden of HIV, yet EHE lacks specific mention of correctional settings for intervention. We conducted a survey study of current HIV and HCV care practices in prisons and jails serving EHE hotspots.

Figure 1

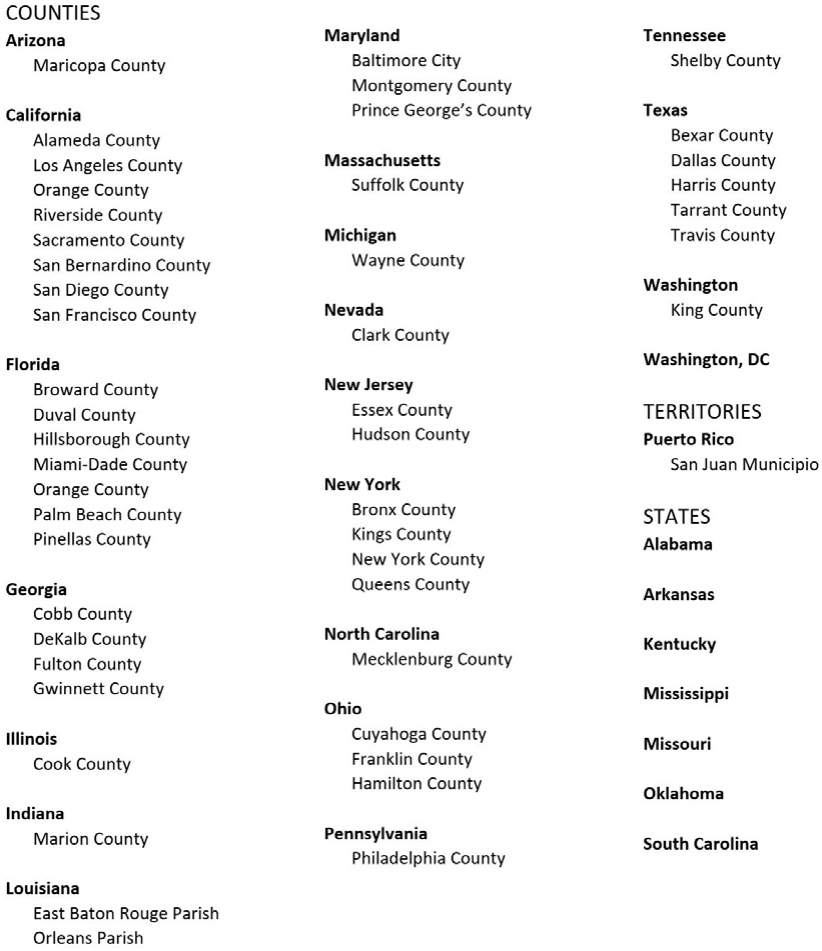

Priority jurisdictions for the “Ending the HIV Epidemic” Initiative which include counties, rural states, and territories with the highest HIV burden, together accounting for more than 50 percent of new HIV diagnoses in recent years. Source: Division of HIV/AIDS Prevention, Centers for Diseases Control and Prevention, https://www.cdc.gov/endhiv/jurisdictions.html

**Methods:**

An online survey on HIV/HCV testing, prevention, treatment, and surveillance was sent to Medical Directors or designees at 26 state prison systems and 37 county or city jails serving EHE hotspots in Spring 2021.

**Results:**

Twenty-five responses were received (10/26 prisons, 15/37 jails) for an overall response rate of 40%. Routine HIV testing, defined as testing offered to all persons without known infection, was conducted in 76% of facilities (9/10 prisons, 10/15 jails), with policies of “opt-out” in 44% (5/10 prisons, 6/15 jails), “opt-in” in 20% (2/10 prisons, 3/15 jails), and “mandatory” in 12% of facilities (2/10 prisons, 1/15 jails). Most facilities (80%) provided HIV testing upon inmate request. For HIV prevention, education programs and/or treatment for opioid-use disorder was available in 76% of facilities, but PrEP and condoms were only available in 24% and 16%, respectively. All facilities reported providing antiretroviral therapy and 88% provided a short (3- to 30-day) supply upon discharge. Routine testing for HCV was conducted in 52% of facilities (7/10 prisons, 6/15 jails), with policies of “opt-out” in 36% (5/10 prisons, 4/15 jails), “opt-in” in 12% (1/10 prisons, 2/15 jails), and “mandatory” in one prison. Most facilities (80%) provided HCV testing upon inmate request. In 8/10 prisons and 6/15 jails, HCV treatment with direct-acting antivirals was continued if initiated prior to incarceration. Treatment for new diagnoses of HCV was less common (16-44%) and depended on expected length of incarceration.

**Conclusion:**

In prisons and jails serving HIV “hotspot” regions, critical opportunities for improved HIV and HCV testing, treatment, prevention, and linkage-to-care services remain. Given these findings, we support the broader inclusion of the justice system as an integral component of the EHE initiative.

**Disclosures:**

**All Authors**: No reported disclosures

